# Exploring the digital technology preferences of teenagers and young adults (TYA) with cancer and survivors: a cross-sectional service evaluation questionnaire

**DOI:** 10.1007/s11764-017-0618-z

**Published:** 2017-06-20

**Authors:** Esha Abrol, Mike Groszmann, Alexandra Pitman, Rachael Hough, Rachel M Taylor, Golnar Aref-Adib

**Affiliations:** 1 Highgate Mental Health Centre, Camden and Islington NHS Foundation Trust, Dartmouth Park Hill, N19 5NX London, UK; 2 Teenage & Young Adult Psych-Oncology Team, Psychological Medicine Dept. Paediatric & Adolescent Division, University College London Hospital NHS Foundation Trust; UCL Undergraduate Medical School, 6th Floor, 250 Euston Road, UCLH, NW1 2BU London, UK; 3 Macmillan Cancer Psychological Support Team, St George’s University Hospitals NHS Foundation Trust, London, UK; 4 Division of Psychiatry, University College London, 149 Tottenham Court Road, W1T 7NF London, UK; 5 Haematology and Stem Cell Transplantation, University College London Hospital NHS Foundation Trust, London, UK; 6 University College London Hospital NHS Foundation Trust, London, UK; 7 Camden and Islington NHS Foundation Trust, St Pancras Hospital, 4th Floor, East Wing, 4 Saint Pancras Way, NW1 0PE London, UK

**Keywords:** TYA, AYA, Teenagers, Young adults, Survivorship, Digital communications, Service development, Technology, Psycho-oncology

## Abstract

**Purpose:**

Digital technology has the potential to support teenagers and young adults (TYAs) with cancer from the onset of their disease into survivorship. We aimed to establish (1) the current pattern of use of TYA digital technologies within our service-user population, and (2) their preferences regarding digital information and support within the service.

**Methods:**

A cross-sectional survey was administered as a paper and online self-completed questionnaire to TYAs aged 13–24 accessing outpatient, inpatient, and day care cancer services at a regional specialist centre over a 4-week period.

**Results:**

One hundred two TYAs completed the survey (55.7% male; 39.8% female; 83.3% paper; 16.7% online; mean age 18.5 years [SD = 3.51]). Of the TYAs, 41.6% rated the importance of digital communication as “essential” to their lives. Half (51.0%) kept in contact with other patients they had met during treatment, and 12.0% contacted patients they had not met in person. Respondents wanted to receive clinical information online (66.3%) and use online chat rooms (54.3%). Future online services desired included virtual online groups (54.3%), online counselling or psychological support (43.5%), and receiving (66.3%) and sharing (48.9%) clinical information online.

**Conclusions:**

Young people with cancer are digital natives. A significant subgroup expressed a desire for digital resources from oncology services, though existing resources are also highly valued. Digital resources have potential to improve patient experience and engagement.

**Implications for cancer survivors:**

There is considerable scope to develop digital resources with which TYAs can receive information and connect with both professionals and fellow patients, following diagnosis, through treatment and survivorship.

## Introduction

### TYA cancer patients and survivorship

Each year, there are approximately 2300 new cases of cancer in teenagers and young adults (TYA) aged 15–24 years in the UK. Data from 2001 to 2005 show that more than 80% survive the disease for at least 5 years in the UK [1–3], and similarly, US data show overall 5-year survival rates for adolescents (15–19 years) of 77% from 1985 to 1994 [4]. These young people are faced with a life-changing diagnosis at a critical stage of social and emotional development, an experience unlikely to be shared by many of their peers [5, 6].

A “cancer survivor” definition was agreed at the UK National Survivors Conference (2008) to be “anyone living following a cancer diagnosis”, in other words, from day 2, the day after diagnosis [7, 8]. Whilst coping with the initial diagnosis and treatment is a major challenge for a young person to overcome, the longer-term psychological impact of survivorship carries an additional burden [9]. TYAs are likely to need tailored psychosocial support to address survivorship issues, and patient empowerment is recognized as important in optimizing their ability to cope with survivorship [10, 11]. Such issues include negative body image, fear of disease recurrence, and facing loss of fertility [10]. Young people with cancer are also susceptible to psychological difficulties such as stress, anxiety, depression [10, 12, 13], and suicidal behaviour [14], particularly in the first year after diagnosis [15], but with risk remaining elevated even in long-term survivors [14].

### The role of digital technology in TYA cancer patients and survivors

Young people are “digital natives” who have grown up using the Internet [16]; 82% of young people aged between 13 and 24 access the Internet daily [17], and in 2013, young people aged 15–24 were shown to spend an average of 40 h online per month [18]. Cancer survivors access the Internet at a lower rate than the general population, but once on the Internet, they are more likely to use it for health-related purposes [19] or as a route to address their unmet needs, resulting in increased knowledge, treatment adherence, dissemination of symptoms distress, and ultimately, improvement in the quality of life [20]. Although the value of face-to-face support from clinicians and allied healthcare professionals is of unrequited importance, there is clearly an opportunity to respond to the evolving digital needs of at least a subgroup of TYAs with cancer [21].

The volume of information on the web can be overwhelming, and some sites contain misleading or irrelevant information. It is important for patients to discuss information gathered online with their care providers within the safety of a multidisciplinary team (MDT) as part of the shared decision-making process. The national scoping exercise, More Than My Illness (2010) [22] undertaken by CLIC Sargent,^1^ reports that “every young person should be well informed, empowered and supported to make choices” (point 3.3, p22), and refers to the National Cancer Survivorship Initiative [23], a coordinated and standardized digital information resource that will be made available to all young people. Aiming High for Young People [24] emphasizes the importance of empowerment, suggesting that “when young people have the opportunity to influence services they are more likely to find them attractive and to access and benefit from them”.

A recent critical review concluded that the implementation of supportive technology for TYAs with chronic illness is hindered by the poor quality of current evidence, the lack of involvement of TYAs in research studies, and a poor understanding of their specific needs [25]. Children and young people’s perception of technology and how it is used do not necessarily align with the perceptions of health professionals. User involvement has become a central tenet of service development, policy, and research in the UK [26], and working in collaboration with TYAs is key to ensuring the acceptability and feasibility of services [25].

Currently, clinical guidelines in the UK highlight the importance of directing TYAs with cancer to evidence-based and age-appropriate sources of reliable information online [10, 27]. These include local hospital websites and a number of largely charity and service-user driver websites that are poorly integrated. These services have been summarized in Box 1. The experience of clinicians at UCLH delivering care to TYAs with cancer is that many would appear to benefit from psychological support, but are often unwilling or unable to accept this for a number of reasons [25]. Patients having intensive radiotherapy, chemotherapy, or surgical treatment for cancer are often reluctant to spend further time in hospital than they need to, or to have yet more clinical professionals involved in their care. During treatment, they are sometimes not physically well enough to access psychological support, or prefer not to travel long distances for this. After treatment, they may be hesitant to return to hospital [28].

Patients and their families have often made enquiries about accessing both professional and peer psychological support remotely online. They have mentioned this preference for the following reasons: the anonymous nature of this help; its availability from any site, even if neutropaenic; its ease of accessibility and convenience; the general appeal of the digital medium; and the wish to access non-professional peer support, which feels less stigmatizing and more normalizing [25, 29, 30]. Similarly, patients in the UCLH TYACS are known to regularly request access to non-psychological support, in terms of contacting and accessing advice from cancer professionals, getting patient information, and arranging services digitally [25, 29, 30].

Box 1 Websites used to access TYA related support

**Table:** 

Organization	Website link (URL)	Description	Reference
London Cancer	www.londoncancer.org	An integrated cancer system serving north-east and central London and west Essex. The network works with healthcare providers in these areas to deliver comprehensive and seamless cancer care from diagnosis, through treatment, to living with cancer and beyond.	[31]
Teenage Cancer Trust (TCT)	www.teenagecancertrust.org	A UK-based charity providing expert treatment and support.	[32]
Jimmy Teens TV	https://jtvcancersupport.com/	A project for TYAs who have been affected by cancer with video diaries and films sharing experiences of cancer.	[33]
MacMillan Cancer	www.macmillan.org.uk/Cancerinformation/teensandyoungadults	A UK-based charity providing support, events, campaigns to those living with or affected by cancer.	[34]
Youth Health Talk	www.youthhealthtalk.org/Teenage_Cancer	A free reliable source of information about health issues through sharing real-life stories and experiences. Partnership between a charity called DIPEx and The Health Experiences Research Group (HERG), The University of Oxford Nuffield Department of Primary Care.	[35]
Shine Cancer Support	www.shinecancersupport.co.uk	A UK-based charity supporting younger adults living with cancer and beyond. Provides tailored information and peer support through a range of activities including lunches, drinks evenings, online networking, etc.	[36]
Barts and the London Kids	https://bartscharity.org.uk/	Hospital website offering support and services.	[37]
CLIC Sargent	www.clicsargent.org.uk	Provides vital emotional, practical, and financial support to young cancer patients and families during and after treatment.	[38]
London Sarcoma	www.londonsarcoma.org	One of the largest sarcoma services in Europe with international reputation for providing the highest quality of care to patients with sarcoma.	[39]

The Wellcome Trust, a key UK funding body, highlighted the importance of integrating digital technologies into studies to “drive innovation, facilitate engagement of young people, and deliver interventions” in lessening the burden of mental health problems which account for 13% of all the years lived with any disability [29]. To ensure that any future digital interventions are desired, feasible, usable, sustainable, and acceptable, it is essential that we first establish the patterns of use and preferences of young people in the TYA population, who would seem most likely to benefit from digital interventions.

### Aims of the study

The aims of this study are shown below:

1. To establish the current pattern of use of TYA digital technologies within our service-user population
2. To establish the preferences regarding digital information and support within the service

Our objective was to use the findings of this descriptive study as a platform to inform future service developments integrating digital resources.

## Methods

### Study design and participants

We conducted a cross-sectional study of TYAs receiving cancer treatment at University College London Hospitals (UCLH) NHS Foundation Trust, Teenage and Young Adult Cancer Service (TYACS). Inclusion criteria were all TYA patients aged 13–24 years receiving treatment or undergoing follow-up at UCLH, including outpatients, oncology ward inpatients, and day care patients, during a 4-week data collection period. There is poor consensus internationally on the age definition of TYAs. Cancer Research UK defines TYAs as 15–24, whereas the US National Cancer Institute defines adolescents and young adults (AYAs) as 15–39. We chose the lower age limit of 13–24, which is accepted by the Teenage Cancer Trust (TCT), UK, and reflects the national clinical services providing treatment, support, and guidance to TYAs with cancer in the UK, and specifically the age range treated within UCLH TYACS [31]. This was therefore perceived an acceptable adjustment to aid the development of future services at UCLH TYACS and provides more information on a younger age group. It is conventionally accepted that TYAs can be divided into two distinct groups: adolescents, aged 13–18 years, and young adults, aged 19–24 years [27]. This distinction has been adopted by the authors as a part of a subgroup analysis.

All patients fulfilling the inclusion criteria were invited to take part in an anonymized survey to help clinicians improve the quality of digital resources available to TYAs with cancer. Those who agreed to take part in this study were offered a paper or online version of the questionnaire. The online version was hosted by a commercial survey company (SurveyMonkey®) on a closed survey site. The background and purpose of the survey, and information regarding collaborators (UCLH TYACS, London Cancer), was provided for both written and online versions of the survey.

Patients were offered paper versions of the survey on arrival at reception in the outpatient clinic by the receptionist and the clinic nurse. Staff nurses, Clinical Nurse Specialists, and CLIC Sargent Social Workers also disseminated the survey to their allocated patients, offering paper copies to patients attending clinic, as well as day care patients and inpatients on the oncology wards, and sending the electronic survey link by email. Participants choosing the paper version returned completed questionnaires via deposit boxes located at the reception (shared by the TYACS outpatient clinic and day care), by postal mail, or by scanned emailed copy. These options allowed participants to complete the questionnaire in their own time and preferred location.

### The University College London Hospital Teenage and Young Adult Cancer Service

UCLH TYACS is one of 20 specialist Principle Treatment Centres (PTC) in the UK for the diagnosis, monitoring, and management of 13–25 year olds with cancer. It offers a multidisciplinary psycho-oncology service to patients and their families alongside surgical and medical treatments for cancer. UCLH TYACS was the first specialist unit for teenagers and young adults in the world, established in 1990, and the largest TYA Cancer Service in Europe, treating about 270 new cases a year (about 15% of all TYA cancers in the UK) [40]. The catchment area for the service covers a very wide geographical area with a population of 6.7 million people. All patients aged 13–18 years are referred to PTC whilst 19–24 year olds are offered a choice between referral to the PTC or a “Designated Hospital” closer to home. Although the conventional age range for TYAs is 15 to 24, our study included teenagers from the age of 13 to reflect UCLH TYACS local service provision [31].

### Procedures

The questionnaire (Box 2) consisted of 15 self-administered questions covering domains such as socio-demographic characteristics, current use of digital communications, patients’ experiences of online cancer resources, and suggestions regarding needs for digital support. We collected information on basic demographic characteristics (gender, race, religion) as convention in order to assess whether our sample differed from the background population.

Box 2 Survey questionnaire

**Table:** 

**At the moment…** 1. What do you use to chat to friends and family? a. Phone b. Text c. Facebook d. Blackberry Messenger (BBM) e. Twitter f. Skype g. Email h. Whatsapp i. Other (please specify): _______________________________ *(Respondents were asked to select all those that apply.)* 2. What do you use to go online? a. Smartphone b. PC c. Laptop d. Tablet (e.g. iPad) e. Games console f. Other (please specify): _______________________________ *(Respondents were asked to select all those that apply.)* 3. Do you keep in touch with people you have already met who have or had cancer? e.g. people you met in hospitals, clinics, online groups, etc. *(Respondents were asked to select “yes” or “no”. If the latter was selected, they skipped question 4.)* 4. If yes, how do you keep in touch? a. Phone b. Text c. Facebook d. Blackberry Messenger (BBM) e. Twitter f. Skype g. Email h. Whatsapp i. Other (please specify): _______________________________ *(Respondents were asked to select all that apply.)* 5. Do you ever contact people you haven’t ever met before, who have or had cancer? *(Respondents were asked to select “yes” or “no”. If the latter was selected, they skipped question 6.)* 6. If yes, how do you contact them? a. Phone b. Text c. Facebook d. Blackberry Messenger (BBM) e. Twitter f. Skype g. Email h. Whatsapp i. Other (please specify): _______________________________ *(Respondents were asked to select all those that apply.)* 7. How do you find out about your hospital and the treatments you are getting? a. Information from professionals b. Leaflets c. Books d. Websites e. Friends/family f. Other (please specify): _______________________________ *(Respondents were asked to select all those that apply.)* 8. Have you used the website of: a. Your local hospital b. UCLH c. The MacMillan Cancer Centre d. London Cancer e. Other (please specify): _______________________________ *(Respondents were asked to select all those that apply.)* 9. How do you try to find out about your cancer and what can be done about it? a. Information from professionals b. Leaflets c. Books d. Websites e. Friends/family f. Other (please specify): _______________________________ *(Respondents were asked to select all those that apply.)* 10. How important is digital communication to your life? *(Assessed on a 7-point Likert-style scale ranging from “Not at All” to “Essential”.)* 11. These are some of the websites we have links to. Please tick any you have looked at and rate them out of 7 (see Box 2 for descriptions): a. www.londoncancer.org [31] b. www.teenagecancertrust.org [40] c. https://jtvcancersupport.com [32] d. www.macmillan.org.uk/Cancerinformation/teensandyoungadults [34] e. www.youthhealthtalk.org/Teenage_Cancer [38] f. www.shinecancersupport.co.uk [41] g. https://bartscharity.org.uk [42] h. www.clicsargent.org.uk [43] i. www.londonsarcoma.org [44] j. Other (please specify): _______________________________ *(Each rated using a 7-point Likert-style scale ranging from “Useless” to “Excellent”.)* ** In the future…** 12. What would you like to have available? a. Virtual online groups to chat to other young people who have to deal with cancer? b. Counselling or psychological support online? c. Receive information about your clinical condition, treatments, or sources of support? d. Able to share personal clinical information with professionals online? e. Parents to have access to your online clinical information? *(Respondents were asked to select “yes” or “no”.)* 13. Which professionals would you want to contact digitally, and how would you like to contact them? *(Respondents were asked fill in a table with two columns: “professionals I’d like to contact” and “I would want to contact them by”.)* 14. What other services would be good to have online? *(Respondents filled in a white space answer box.)* 15. Any other ideas or comments? *(Respondents filled in a free text box.)*	

At the time of conducting this survey, the authors were not aware of validated tools for the assessment of digital technology use, but instead used an unvalidated measure of digital technology use in TYAs with cancer. Our questionnaire was developed in collaboration with the North Thames Children’s Cancer Network Coordinating Group (CCNCG), UCLH TYA cancer service staff, and research staff with an interest in TYA Cancer and associated with UCLH: this team consisted of oncologists, haematologists, oncology specialist nurses, allied health professionals working in oncology, psychiatrists, and psychologists. The network advised on important themes to include in the questionnaire, as well as appropriate wording. The prototype questionnaire included closed questions with multiple-choice responses and Likert-type rating scales to collect quantitative data, and open questions with free-text responses. This version was reviewed by the UCLH TYA cancer senior management group, and revised. Revisions included adjustments to language, flow, demographic details collected, and websites specified, in order to evaluate our service better. The questionnaire was piloted with 20 TYAs under the care of UCLH TYACS. Minor amendments were made based on responses and feedback, but no formal changes to content were indicated.

### Ethical approval

As this study was regarded as a service improvement project, we were not required to gain formal approval from an ethics committee. To ensure that participants gave informed consent, we provided full written information about the survey. Completion of the survey was felt to imply informed consent.

### Statistical analysis

Data from the paper versions of the questionnaire (*n* = 85) were entered manually into the SurveyMonkey® link so that it was merged with the online responses (*n* = 17), and all data were imported into Microsoft Excel, 2013. All analyses were conducted using SPSS version 21. Descriptive statistics, such as frequency distributions, were used to describe and summarize the characteristics of the sample. Categorical data were described using chi-squared tests, and continuous data were described using independent *t* tests. It is conventionally accepted that TYAs can be divided into two distinct groups: adolescents, aged 13–18 years old, and young adults, aged 19–24 years [27]. This distinction has been adopted by the authors as a part of a subgroup analysis.

## Results

### General characteristics (Table 1)

A total of 102 responses were received; the majority elected to express their views on paper (83.3%) versus online (16.7%). Reasons for this include the feasibility, practicality, and acceptability of completing the questionnaire as a hard copy whilst waiting for their outpatient clinic appointment. It was not compulsory to answer all questions, and the number of missing responses is shown in Table 2. Questions requiring rating or free text were most likely to be missed. We do not have an accurate denominator as the total number of patients approached to participate was not recorded. However, staff were encouraged to approach all patients attending the unit during the recruitment period. There were 239 patient attendances during this time, giving an approximate response rate of 42.7%. The general demographic characteristics of the sample are found in Table 1. The majority of respondents stated their gender as male (49/88, 55.7%) versus female (35/88; 39.8%). The mean age of respondents was 18.5 (SD = 3.51).

**Table 1:** General characteristics (*n* = 102)

Category	Parameter	Number	Percentage
Gender (*n* = 88)	Male	49	55.7%
	Female	35	39.8%
	Prefer not to say	4	4.5%
	Missing data	14	13.7%
Race (*n* = 88)	White	45	51.1%
	Asian/Asian-British	16	18.2%
	Black/Black-British	11	12.5%
	Chinese	1	1.1%
	Mixed	5	5.7%
	Prefer not to say	5	5.7%
	Other	5	5.7%
	Missing data	14	13.7%
Religion (*n* = 88)	Christian	29	33.0%
	Atheist	14	15.9%
	Muslim	9	10.2%
	Hindu	6	6.8%
	Jewish	2	2.3%
	Other	4	4.5%
	Agnostic	7	8.0%
	Prefer not to say	17	19.3%
	Missing data	14	13.7%

**Table 2:** Digital technology preferences of the cohort (*n* = 102)

Category	Parameter	Number	Percentage
What do you use to chat to friends and family? (*n* = 100)	Phone	82	82.0%
	Text	81	81.0%
	Facebook	76	76.0%
	Whatsapp	56	56.0%
	Skype	40	40.0%
	Twitter	36	36.0%
	Email	30	30.0%
	BBM	11	11.0%
	None	0	0.0%
	Other	5	5.0%
	Missing data	2	2.0%
What do you use to go online? (*n* = 100)	Laptop	79	79.0%
	Smartphone	72	72.0%
	Tablet	36	36.0%
	PC	30	30.0%
	Games console	18	18.0%
	Other	2	2.0%
	None	1	1.0%
	Missing data	2	2.0%
Do you keep in touch with people you have already met who have or had cancer? (*n* = 100)	No	49	49.0%
	Yes	51	51.0%
	Missing data	2	2.0%
If yes, how do you keep in touch? (*n* = 51)	Text	35	68.6%
	Facebook	25	49.0%
	Phone	20	39.2%
	Whatsapp	9	17.6%
	Twitter	7	13.7%
	Email	6	11.8%
	Skype	4	7.8%
	Other	4	7.8%
	BBM	1	2.0%
	Missing data	51	50.0%
Do you keep in touch with people you haven’t ever met before, who have or had cancer? (*n* = 100)	No	88	88.0%
	Yes	12	12.0%
	Missing data	2	2.0%
If yes, how do you contact them? (*n* = 10)	Text	2	20.0%
	Facebook	3	30.0%
	Phone	2	20.0%
	Whatsapp	0	0.0%
	Twitter	1	10.0%
	Email	1	10.0%
	Skype	1	10.0%
	Other	5	50.0%
	BBM	0	0.0%
	Missing data	92	90.2%
How do you find out about your hospital and the treatments you are getting? (*n* = 97)	Information from professionals	86	88.7%
	Leaflets	39	40.2%
	Books	6	6.2%
	Websites	34	35.1%
	Friends/family	18	18.6%
	Other	2	2.1%
	Missing data	5	4.9%
Have you used any of the following websites? (*n* = 97)	Your local hospital	9	9.3%
	UCLH	40	41.2%
	The MacMillan Cancer Centre	41	42.3%
	London Cancer	3	3.1%
	Other	2	2.1%
	Missing data	5	4.9%
How do you try to find out about your cancer and what can be done about it? (*n* = 93)	Information from professionals	77	82.8%
	Leaflets	41	44.1%
	Books	12	12.9%
	Websites	41	44.1%
	Friends/family	19	20.4%
	Other	5	5.4%
	Missing data	9	8.8%
In the future what would you like to have available? (*n* = 92)	Virtual online groups to chat to other young people who have to deal with cancer	50	54.3%
	Counselling or psychological support online	40	43.5%
	To receive information about your clinical condition treatments, sources of support	61	66.3%
	To be able to share personal clinical information with professionals online	45	48.9%
	Parents to have access to your online clinical information	43	46.7%
	Missing data	10	9.8%
Which professionals would you like to contact digitally and how? (*n* = 33)	Doctors	14	42.4%
	Clinical Nurse Specialists/Nurses	11	33.3%
	Psychologists/counsellors	3	9.1%
	Social worker	1	3.0%
	Radiographer	1	3.0%
	None	1	3.0%
	Missing data	69	67.6%
How would you like to contact the above individuals? (*n* = 33)	Email	21	63.6%
	Chat/forums	5	15.2%
	Facebook	1	3.0%
	Skype	1	3.0%
	Text	2	6.1%
	Phone	3	9.1%
	Other	2	6.1%
	Missing data	69	67.6%
How important is digital communication to your life (*n* = 93)	Essential (7/7)	42	45.2%
	6.00	24	25.8%
	5.00	15	16.1%
	4.00	10	10.8%
	3.00	2	2.2%
	2.00	0	0.0%
	Not at all (1/7)	0	0.0%
	Missing data	9	8.8%

### Digital technology preferences (Table 2 and 3, Fig. 1)

**Fig. 1:**
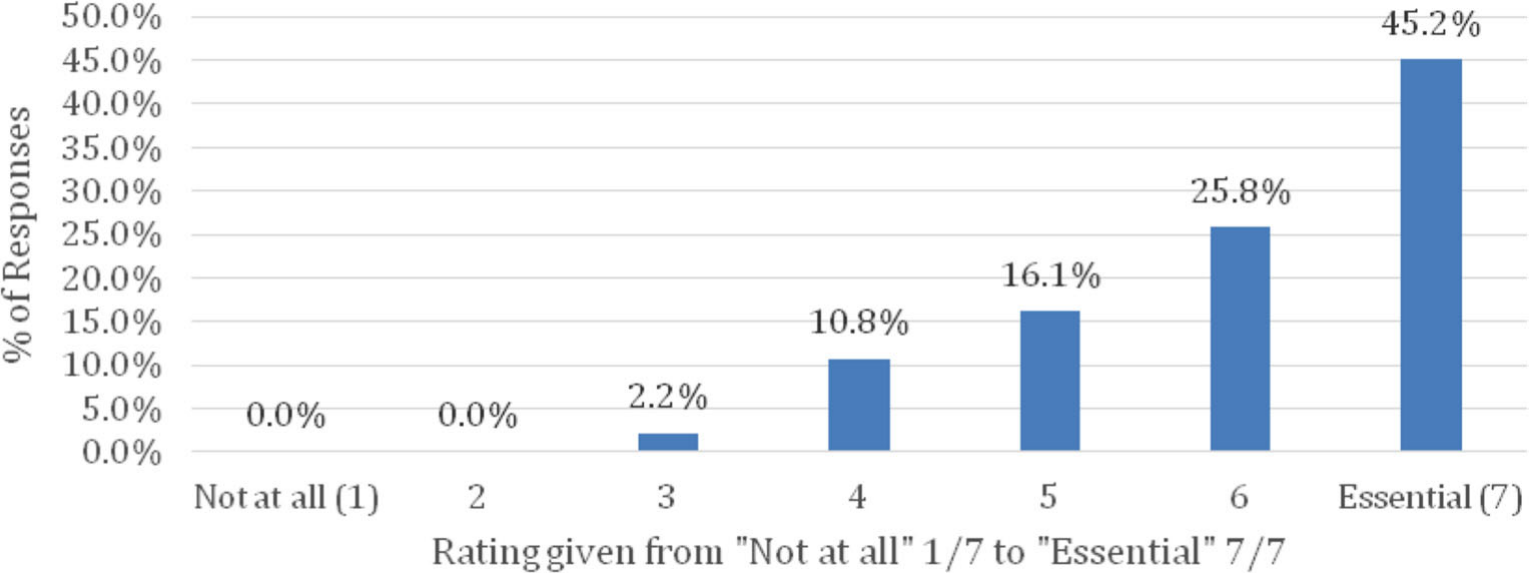
How important is digital communication to your life? (*n* = 93)

TYAs were asked to rate the question “How important is digital communication to your life?” from “essential” (7/7) to “not at all” (1/7). Of the TYAs, 41.6% (42/93) rated this maximally (“essential”, 7/7), with an average rating of 6.01 (Fig. 1). The remainder elected 6/7 (24/93; 25.8%), 5/7 (15/93, 16.1%), 4/7 (10/93; 10.8%), and 3/7 (2/93, 2.2%), with no respondents selecting 2/7 or 1/7 (“not at all”). There were nine missing responses for this question (8.8%).

**Table 3:** How would you rate the following websites available to you? (*n* = 60)

Rank	Website	Median rating (on scale of 1—useless, to 7—excellent)
1	www.teenagecancertrust.org	6.04
2	www.macmillan.org.uk/	5.9
3	www.clicsargent.org.uk	5.9
4	www.londoncancer.org	5.27
5	www.londonsarcoma.org	5.25
6	www.bartsandthelondonkids.nhs.uk	5.25
7	jimmyteens.tv	5.2
8	www.shinecancersupport.co.uk	4.57
9	www.youthhealthtalk.org/Teenage_Cancer	4.33
10	Other	2
Missing data	42/102

Table 2 depicts TYA preferences for digital communication methods. Half of the participants (51/100; 51%) reported having contacted patients with current or remitted cancer who they met face to face at various stages of their cancer treatment, largely by means of text messages (35/51; 68.6%), Facebook (25/51; 49.0%), or phone call (20/51; 39.2%). A smaller subset of participants (12/100; 12.0%) had made contact with patients with current or remitted cancer who they had never met before using the following methods in order of preference: Facebook, text message, and phone call. Other methods included Snapchat and FaceTime. The majority of TYAs reported finding out information about their hospital and treatment from professionals (86/97, 88.7%), leaflets (39/97, 40.2%), and websites (24/97, 35.1%). Similarly, when it came to finding out about their cancer and what could be done about it, the majority of TYAs reported consulting professionals (77/93, 82.9%). Of websites listed in the survey questionnaire, those most commonly used by respondents were those of Macmillan Cancer Centre (a nationwide voluntary sector organization) (41/97; 42.3%), University College London Hospitals (UCLH) (40/97; 41.2%), with smaller proportions using those of their local hospital (9/97; 9.3%), and London Cancer (a local networking organization providing information and support to cancer professionals and patients) (3/97; 3.1%).

Table 3 depicts average (mean) ratings of websites recommended to TYAs with cancer through the UCLH TYACS facility. Of the websites listed, those rated as excellent (7/7 on a Likert-type scale) included sites from three nationwide voluntary sector organizations: Teenage Cancer Trust (TCT) [32] (25/60, 41.7%), MacMillan Cancer Support [34] (12/60; 20.0%), and CLIC Sargent [38] (11/60; 18.3%) (Table 3). These organizations (Box 1) provide emotional, practical, or financial advice; social support; and non-medical supportive treatment.

### Preferences for future services (Table 2)

Future online services desired included virtual online groups (50/92, 54.3%), online counselling or psychological support (40/92, 43.5%), and receiving (61/92, 66.3%) and sharing (45/92, 48.9%) clinical information online from professionals.

TYAs wanted to contact doctors (14/33, 42.4%), nurses (11/33, 33.3%) and psychologists (3/33, 9.1%) in majority, largely via email (21/33, 63.6%) or forums (21/33, 63.6%). This data should be interpreted with caution as there were 69 (67.6%) missing responses.

In free-text responses (*n* = 24), TYAs suggested practical peer support modalities such as online chat rooms, symptoms checking facilities, a youth ambassador program, group events and games sections for the websites, information resources to raise awareness for fellow teenagers, possibly including anonymous case histories, and a Frequently Asked Questions (FAQ) service. They also suggested having online access to blood test results, medication charts, and information on side effects, as well as an online appointment booking service.

### Subgroup comparison of responses from two patient age groups; from adolescents (13 to 18) and young adults (19 to 24) (Table 4)

Adolescents (13 to 18 years) were compared to young adults (19 to 24 years). A larger proportion of young adult TYAs (aged 19–24: *n* = 40) compared with adolescent TYAs (13–24; *n* = 44) kept in contact with patients they had not met before (6.8% [13–18] versus 22.5% [19–24]). Regarding future preferences, young adult TYAs were significantly more likely to express a preference for counselling or psychological support online (57.5%) than younger TYAs (31.9%) (*p* = 0.032) and to share their clinical information with professionals online (34.1% [13–18] versus 60.0% [19–24]; *p* = 0.031) (Table 3).

**Table 4:** Suggested future services by age-group

Parameter	Number (13–18)	Percentage	Number (19–24)	Percentage	*p* value
Virtual online groups to chat to other young people who have to deal with cancer	20	45.5%	26	65.0%	0.115
Counselling or psychological support online	14	31.8%	23	57.5%	0.032^a^
To receive information about your clinical condition, treatments, sources of support	26	59.1%	32	80.0%	0.067
To be able to share personal clinical information with professionals online	15	34.1%	24	60.0%	0.031^a^
Parents to have access to your online clinical information	24	54.5%	19	47.5%	0.538

^a^Statistically significant: *p* < 0.05

## Discussion

### Main findings

Data from our large sample of TYAs with cancer from a UK Principal Treatment Centre show that TYAs have active digital lives. They are using a variety of healthcare-related digital resources, both those that have been recommended to them by the UCLH TYACS and independent sources. We found that TYAs are using digital technology to maintain relationships with patients they meet during their cancer journey, and that a smaller proportion, approximately 1 in 10, establish new relationships with those they have not met face to face through digital mediums.

TYAs are accessing information about their treatment predominantly from professionals in a face-to-face environment. This is a reassuring and appropriate finding as this is the conventional means by which TYA oncology care is delivered by the multidisciplinary team whether or not TYAs choose to engage. This emphasizes the importance of a good doctor-patient relationship in the oncology clinic. Although TYAs are offered a second opinion if they are uncertain or unhappy with their face-to-face treatment, further study is needed to investigate whether this is a desired alterative.

One in five TYAs prefer to receive information about their cancer and treatment outside of the face-to-face environment, potentially through adjunctive digital supportive technologies. Our subgroup analysis of adolescents (13–18) versus young adults (19–24) showed that young adults (19–24 years) tended to have a preference for online counselling and receiving clinical information online. Reasons for this may include increasing independence, resilience, more breadth of experience in the digital world, and greater confidence discussing clinical matters online. Although useful as a guide, a larger sample, and more fine-grained age categories, is necessary in order to explore any differences in the digital preferences of these two groups in detail. These results suggest that (a) patients should be offered the choice of face-to-face interaction and/or an alternative, and (b) alternatives to face-to-face should be made freely available. Alternatives, or adjuncts, to face-to-face encounters have been suggested by TYAs to include receiving and sharing clinical information from professionals online, online forums with professionals and fellow patients, and online counselling.

### Findings in the context of other studies

Our findings are consistent with existing literature on general population TYA samples in which over 90% of TYAs [41, 42] report regular access to the Internet and, specifically, to Facebook, YouTube, Twitter, and Jimmy Teens TV (JTV) (online sharing of short films made by TYAs who have been affected by cancer). The proportion of TYAs with cancer reporting laptop, smartphone, and tablet use were similar to previous studies, at approximately 70–90% [42].

Despite this level of Internet use, the literature suggests infrequent communication with strangers online [41], as demonstrated in the present study. Whilst a minority of TYAs with cancer are using the Internet to form new relationships, the majority are using it as an accessible medium to communicate and facilitate the maintenance of pre-existing relationships and traditional social interaction. A UK study highlighted that talking to others who had been through a similar cancer experience through face-to-face or online contact was considered a very useful source of online support [42]. This may explain why TYAs are talking to those they have not met through online mediums.

In terms of future service improvements, our findings are supported by Moody et al. [42] who surveyed and interviewed young cancer survivors. They found that, as well as an abundant need for social support through peer interaction, TYAs wanted clinical self-management tools online. This demonstrates the importance of sharing feelings and experiences online—usually with peers/family/fellow patients—whilst deriving information about illness and treatment from professionals.

TYAs surveyed showed a preference for suggested future online interventions (virtual online groups, counselling online, receiving and sharing clinical information online) in proportions of 43.5–66.3% (Table 2). This represents a dichotomy between respondents who wanted to engage with supportive digital mediums and those who did not. This split in preference is very interesting and has been shown in previous audits, peer-reviewed studies, service surveys within UCLH TYACS [42], and anecdotal observations by professionals in other centres nationally [43, 44]. Reasons for this may include the influence of parental preference, especially in adolescents, a desire in a significant subgroup to separate their clinical lives from their social lives [42], and finally, convention. Another explanation based on observations and experience of clinicians working at UCLH TYACS is that a subgroup of patients respond to their illness by actively seeking available support and engaging and connecting with services and other patients: an “accepting” or help seeking style. Another subgroup seem to find their illness overwhelming and too distressing and tend to avoid taking up the services offered. The latter are likely to isolate themselves, seeking to return to their “normal life”, with minimal thinking or talking about their cancer experience: a “denial” or help avoidance style. It is possible that the help avoidant are less likely to respond to adjunctive digital supportive interventions. Our survey was not designed to elucidate or identify this potential pattern of responding to illness, and future studies are needed to explore this more directly.

Clinical experience suggests that more “avoidant” patients are less likely to adhere to treatment plans; more difficult to engage; more likely to miss appointments, scans, treatments; and more challenging to look after. Logically, they may be likely to have worse clinical and psychosocial outcomes accordingly. Novel and innovative approaches to working with this subgroup are needed, to find acceptable and accessible ways to offer care to those that prefer to avoid “embracing” their illness and all that surrounds it [43]. Studies that have explored different coping styles (e.g. acceptance, denial, fighting stance [27, 45, 46]) have found outcome patterns that are compatible with this finding. It has also been suggested that the Internet can have an isolating effect by promoting feelings of anxiety and loneliness, although this was disproved by a 2004 US study [41] suggesting that this is an outdated theory. Further clarification, in the form of qualitative analysis, is needed to validate this phenomenon, although compliance and uptake in TYAs may be an issue when designing studies.

### Strengths and limitations

We accessed a relatively large sample of over 100 TYAs receiving treatment at a Principal Cancer Centre, in the context of approximately 270 new referrals to UCLH TYACS each year, and 2300 new cases of cancer in TYAs per year in the UK [1, 2]. TYAs are typically poorly compliant with methods to understand their needs, but engaged relatively well with this survey [47]. We estimate that approximately half of those approached declined to participate (response rate 42.7%), and acknowledge non-response bias. TYAs are extremely difficult to gain a response from, with 31–38% of 16–24-year olds completing the national cancer patient experience survey in England [47]. We considered the survey a success accordingly, and can assume that the subject reflects the priority that the digital world represents for TYAs. Given that we sampled in a specialist centre, we are likely to have gained a unique and wide-ranging perspective of the digital communication practice of TYAs in and around an urban centre. There is relatively little published information on the digital habits and preferences of TYAs with cancer. This survey has been presented at national conferences [43] and education days [44, 48], and was very well received by professionals working with TYA Cancer, including winning the Lisa Thaxter Award at the TYAC annual education day [44].

Our study has a number of limitations. We were unable to obtain an accurate response rate, but instead relied on an estimate based on the number of clinic encounters (outpatients), the number of inpatients, and the number of day care patients during our study period. This figure should be interpreted with caution as it may be overestimated due to the presence of duplicate clinic attendances. In order to maximize the response rate in this sample, the authors employed certain measures: to keep the survey short and specific to digital preferences and allowing questions to be “skipped” to avoid attrition and response fatigue. In order to improve our response rate further, we could consider providing handheld devices to patients in clinics and on the ward in order to encourage survey completion, use a more interactive survey platform, survey reminder alerts, hold awareness-raising events, and give “freebies” from charity organizations, prize draws, or voucher incentives. Questions with free text responses were most likely to be skipped (“what other services would you like online?”—24/102 responses; “any other ideas/comments?”—10/102 responses), and are therefore not reliably representative of the wider group. This is a consequence of designing an opportunistic questionnaire from first principles, with the intention to appeal to TYAs and guide local service development at the expense of using an internationally validated tool. If running the survey again, it would be useful to run a pilot in order to explore what would make them most likely to engage with questions in the survey.

Further, our sample consists of TYAs attending a Principal Treatment Centre, whilst 50% of TYAs are treated outside of PTCs, suggesting a degree of selection bias and specificity to our service. Other sources of selection bias include the following: respondents were more likely to be those who were interested in digital media; motivated to participate and answer; motivated to engage in future online resources; have English as their first language; and be well-enough to complete the survey. It was observed that inpatients were strikingly less likely to complete the survey. This pattern of non-response in patients on active treatment has been described elsewhere [49]. This pattern is likely to contribute to the observation that TYAs were more likely to complete the questionnaire on paper (83.3% paper versus 16.7% online), as it was more feasible and practical to complete the survey whilst waiting for their outpatient appointments. We are unable to prove this finding as we did not collect data on whether the respondent was an inpatient or outpatient or in day care, or their clinical characteristics. Future studies should aim to record the above confounding factors more clearly to better understand the clinical characteristics and nature of respondents, and attempt to understand in greater depth about patients who decline to complete the survey if this is ethically and practically possible. Future surveys should include patients from all PTCs and specialist TYA Cancer “designated hospitals” nationally. Better still, future studies should capture the views of TYA cancer survivors not receiving specialist long-term surveillance or follow-up, as well as those within specialist centres.

### Clinical and policy implications

The message is clear from our findings that there is considerable scope to develop digital resources with which TYAs can receive information and connect with both professionals and fellow patients, from diagnosis and through treatment and survivorship. Digital adjuncts to their treatment as usual may obviate the need for TYAs to travel large distances as frequently for their routine oncology care, and to access professional and peer-group support remotely. The results of this survey also suggest that existing methods of giving information and support, face-to-face and paper-based, are still preferred by many, and therefore should not be substituted for newer digital resources. As a starting point we hope to develop digital resources tailored to the needs of our patient group, with the input of patient representatives. Patient feedback will be important in refining successive versions of such digital resources.

On the basis of evaluations, we will be able to share our experiences with other treatment centres. Given the central role played by many major voluntary sector organizations, such as Macmillan Cancer Support, CLIC Sargent, and the Teenage Cancer Trust, and the popularity of their websites amongst our sample, it may be feasible for these organizations to take the lead on the national development of digital resources. These may include safe online forums, social events, and gaming events, as suggested here. Again, this will need to be in collaboration with TYA patient representatives to ensure the acceptability of the resources developed and the feasibility of national service implementation. Such resources have the potential to improve clinical outcomes for a significant subgroup of TYA patients in active treatment and those who face issues of survivorship.

## Conclusions

A large proportion of the TYAs with cancer that we surveyed expressed a clear enthusiasm for digital resources with which they can access information and support, connect with fellow patients and healthcare professionals, and gain different perspectives on issues of survivorship. The preliminary results presented here can be used as a platform for TYA services locally, nationally, and globally to develop resources to address these unmet needs. These digital support resources have the potential to improve patient experience and engagement for a large subsection of TYAs treated for cancer.

## Footnotes

1 CLIC Sargent: a UK-based charity organization that provides social support, including vital emotional, practical, and financial assistance to young cancer patients and families during and after treatment
